# Personalized Vestibular Rehabilitation in Persistent Postural–Perceptual Dizziness (PPPD), Unilateral and Bilateral Vestibular Dysfunction: A Comparative Study

**DOI:** 10.3390/jpm16040214

**Published:** 2026-04-13

**Authors:** Pasqualina Maria Picciotti, Rolando Rolesi, Giorgia Rossi, Giuseppe Oliveto, Jacopo Galli

**Affiliations:** 1 Dipartimento di Testa-Collo e Organi di Senso, Catholic University of the Sacred Heart, 00168 Rome, Italy; pasqualinamaria.picciotti@unicatt.it (P.M.P.); jacopo.galli@policlinicogemelli.it (J.G.); 2 Dipartimento di Neuroscienze, Organi di Senso e Torace, Fondazione Policlinico Universitario Agostino Gemelli IRCCS, 00168 Rome, Italy; giorgia.rossi@guest.policlinicogemelli.it (G.R.); g.oliveto92@gmail.com (G.O.)

**Keywords:** dizziness, unilateral vestibular loss, bilateral vestibular loss, PPPD, vestibular rehabilitation, Computed Dynamic Posturography, Dizziness Handicap Inventory

## Abstract

**Background:** In the last few decades, a growing body of evidence has confirmed that vestibular rehabilitation (VR) can improve the symptoms of many unilateral and bilateral vestibular disorders, by facilitating vestibular compensation mechanisms, such as adaptation, substitution, and habituation. However, the usefulness of the vestibular rehabilitation approach in Persistent Postural–Perceptual Dizziness (PPPD) is currently highly debated and unclear. The aim of the present study was to evaluate the efficacy of VR using computerized dynamic posturography in PPPD patients as a single treatment and without other associated psychological or pharmacological therapies. Results were compared with patients with unilateral and bilateral vestibular disfunction, in order to define the role of our rehabilitation model within a framework of personalized therapy for different disorders. **Methods**: We evaluated 44 patients (23 F, 21 M; ranged from 28 to 82 years; mean age 63.72) affected by unilateral vestibular vestibulopathy (UVP) (*n* = 19), bilateral vestibular vestibulopathy (BVP) (*n* = 10) and PPPD (*n* = 15). For each patient, a comprehensive clinical bedside vestibular assessment was carefully performed by expert clinicians, as well as Bithermal caloric tests with videonystagmography (VNG), Video Head Impulse Test (vHIT) and Computed Dynamic Posturography (CDP). The impact of dizziness on quality of life (QoL) was assessed by the Italian Dizziness Handicap Inventory (DHI). All subjects evaluated in this study underwent five rehabilitative therapy sessions in our centre, once a week for 45 min and exercised daily for 30 min at home. All the exercises progressively became more difficult each week. **Results**: Our study showed that vestibular rehabilitation improved quality of life and reduced the level of self-perceived handicap in patients affected by unilateral and bilateral vestibular dysfunction, with significant improvement in DHI total score and posturographic parameters. In PPPD patients, rehabilitation did not significantly modify posturographic performances and the improvement in total DHI score did not reach statistical significance, although a significant change was observed in the functional sub-score. **Conclusions**: Vestibular rehabilitation confirmed its effectiveness in unilateral and bilateral peripheral vestibulopathies. In patients with PPPD, rehabilitation performed with computerized dynamic posturography may reduce subjective handicap and improve some aspects of daily functioning, although the small sample size and the absence of a control group do not allow definitive conclusions about its efficacy.

## 1. Introduction

Vestibular rehabilitation (VR) has been proposed and used for almost 70 years and the evidence of its efficacy and effectiveness has increased in the last 15–20 years. It consists of a series of physical treatments that aim to promote vestibular compensation and/or improve the control of impaired balance in various vestibular and neurological disorders. The literature indicates moderate–strong evidence to support vestibular rehabilitation in the treatment of unilateral and bilateral vestibular hypofunction [1]. Clinicians can offer specific and different exercise techniques, including virtual reality or augmented sensory feedback in order to improve dizziness and imbalance by facilitating vestibular compensation mechanisms, such as adaptation, substitution, and habituation. VR using computerized dynamic posturography has been proposed for the treatment of patients with dizziness and postural impairment [2,3]; however, in the literature, there is a small number of studies describing results in unilateral and bilateral vestibular disorders. Likewise, the usefulness of the vestibular rehabilitation approach in Persistent Postural–Perceptual Dizziness (PPPD) is currently highly debated and unclear.

Staab et al. [4] defined PPPD as a chronic functional vestibular disorder and they describe how this condition manifests as dizziness, instability, or non-rotational vertigo lasting 3 months or more, exacerbated by upright posture, active or passive movement, exposure to complex visual stimuli or moving. Several triggers, like peripheral or central vestibular disorders, different medical illnesses, or psychological distress, can precipitate PPPD and it may be present alone or co-exist with other conditions. First of all, clinical history and then physical examination lead to diagnosis. The Committee for the Classification of Vestibular Disorders of the Bárány Society established diagnostic criteria for PPPD in 2017 [4]. Epidemiological research defined that about 20% of dizziness can be related to PPPD with an important predominance of females (F/M 2:1) [5].

Recent studies suggest that PPPD is characterized by abnormal sensory integration with increased visual dependence and impaired processing of spatial orientation signals. Patients rely on visual and somatosensory input, with reduced flexibility in sensory reweighting for postural control. This maladaptive strategy can lead to spatial disorientation, postural instability, and increased perception of dizziness, especially in the presence of complex visual stimuli. It may also contribute to the development of anxiety [4,6,7]. For this reason, rehabilitative approaches aimed at improving sensory integration and reducing visual dependence could play an important role in the treatment of PPPD.

The aim of the therapy is to reset the system by reducing anxiety and self-control, getting used to provocative factors and promoting automatic movement control until recovery. Understanding the pathophysiology that considers vestibular, postural, cognitive and emotional aspects can allow patients to ameliorate with different therapeutic strategies. As of today, suggested PPPD therapy is mainly multimodal and treatment plans include VR, pharmacological therapy, cognitive–behavioural therapy and psychotherapy [7]. VR combined with cognitive–behavioural therapy, and supported by drugs, can help patients overcome maladaptive balance, recalibrate vestibular systems and improve quality of life. Nevertheless, the use of a combination of treatments leads to not understanding in detail the real effectiveness of each treatment itself. Regarding pharmacological treatment, the more suitable drugs are selective serotonin reuptake inhibitors (SSRIs), followed by serotonin–norepinephrine reuptake inhibitors (SNRIs) [5]. Pharmacological mechanisms need to be clarified and the level of efficacy is currently unclear. According to the indication, the antidepressants are mostly combined with rehabilitation in order to promote compliance. Similarly, cognitive–behavioural therapy and psychotherapy are often should be associatedwith rehabilitation. Finally, few studies on small series have been performed on rehabilitation in PPPD [8]. The results are unclear and, as previously mentioned, are often related to multimodal therapies.

Therefore, the aim of the present study was to evaluate the efficacy of VR using computerized dynamic posturography in PPPD patients as a single treatment and not in association with other psychological or pharmacological therapies. Results were compared with patients with unilateral and bilateral vestibular disfunction, in order to establish the potential role of our rehabilitation model within a framework of personalized therapy for different disorders.

## 2. Materials and Methods

This is a retrospective data collection concerning a study population that underwent vestibular assessments. The patients enrolled in this study underwent vestibular assessment in our centre between June and October 2023. The study was conducted according to the guidelines of the Declaration of Helsinki and approved by the Ethics Committee of Catholic University of the Sacred Heart, Rome (protocol code 0015065/23 and approved on 16 May 2023). We evaluated 44 patients (23 F, 21 M; ranged from 28 to 82 years; mean age 63.72) divided into three groups: unilateral vestibular vestibulopathy (UVP) (*N* = 19), bilateral vestibular vestibulopathy (BVP) (*N* = 10) and PPPD (*N* = 15) (for key clinical findings and overall vestibular assessment outcomes, see Table 1). For diagnosis of different conditions, we considered diagnostic criteria suggested by the Classification Committee of the Bárány Society based on the patient history, bedside examination and laboratory evaluation [4,9,10]. In particular, for unilateral vestibulopathy, we enrolled patients who had not achieved central vestibular compensation three months after the onset of vertigo.

The patient’s history was carefully evaluated by expert clinicians. Clinical bedside vestibular examination consisted of spontaneous nystagmus research, Fukuda test, star-shaped march test and index finger test (for vestibulo-spinal examination), OTR, skew deviation, ocular torsion (counterrolling) and a head tilt (otolithic signs), clinical Head Thrust or Impulse Test (Halmagyi), Head Shaking Test, and Provocative maneuvers (positional test, vibratory test, fistula test). Moreover, Bithermal caloric tests with videonystagmography (VNG ICS AirCal, OTOsuite Vestibular Software Version 120 Build 310, GN Otometrics, Taastrup, Denmark) recording and Video Head Impulse Test (vHIT, ICS Impulse, OTOsuite Vestibular Software Version 120 Build 310, GN Otometrics, Taastrup, Denmark) were also performed in all patients. Finally, postural control was evaluated using Computed Dynamic Posturography (CDP), performed by Equitest, Neurocom Int. Inc., Clackamas, OR, USA. CDP is based on the Sensory Organization Test (SOT), evaluating the contribution of different sensorial afferences in postural control. The SOT was performed with the subject standing on a dual forceplate enclosed by a visual surround as previously described [11]. Data obtained included the composite equilibrium score (ES) showing the weighted average of the different conditions and sensory analysis (SA) showing the contribution of the different sensorial afferences.

The impact of dizziness on quality of life (QoL) was be assessed by the Italian Dizziness Handicap Inventory (DHI) [12]. The DHI comprises a 25-item self-assessment scale designed to evaluate the self-perceived handicap. Total score ranges between 0 and 100 points. The total scores contain physical (DHI-P, 28 points), functional (DHI-F 36 points) and emotional (DHI-E 36 points) sub-scores.

PPPD patients enrolled had not undergone any previous therapy (medical or psychological) and underwent rehabilitation therapy using the vestibular rehabilitation as a single treatment.

Subjects performed five physical therapy sessions in the hospital once a week for 45 min and exercised daily for 30 min at home. Rehabilitation protocols were progressively tailored according to individual patient performance and sensory deficits, supporting a personalized treatment approach.

**Figure 1:**
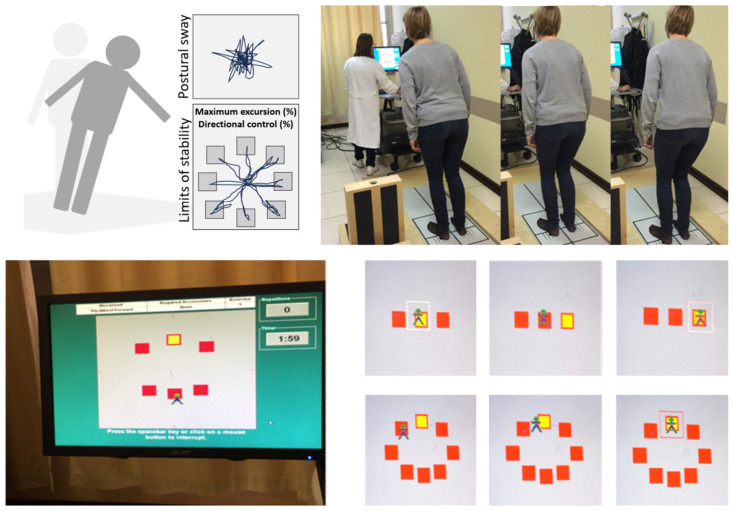
Weight shifting activity.

**Figure 2:**
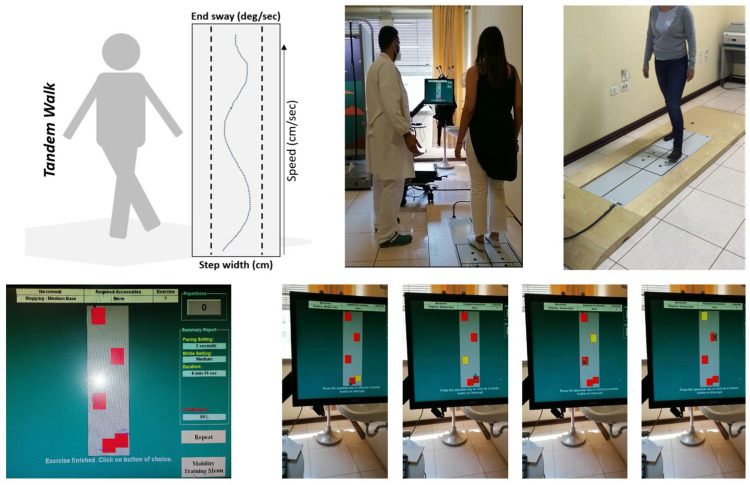
Mobility activity.

Equipment used for instrumental rehabilitation in the hospital included the Neurocom Balance Master Int. Inc., Clackamas, OR, USA, a dual force plate system composed of different cells that detect pressure, connected to a computer and monitor. The system provides a visual representation of the gravity centre of the subject, as previously described [13]. A physician directly supervised each session during the Balance Master training. The Balance Master training protocols have been customized and carried forward by increasing the stability limits and the speed of movement. During the rehabilitation, the subjects were standing and saw on the monitor, at an adequate distance to have a correct aim, several fixed targets that were alternately illuminated (yellow boxes within Figure 1 and Figure 2). Each patient was then asked to hold or shift weight to make sure their centre of gravity representation met the goals presented visually on the monitor. The arrangement of the stimuli varied according to the exercises and the level of complexity of the training. Each exercise lasted from 60 to 120 s. At the end of each session, the system automatically processes a score based on the number of targets reached in the time unit and on the seconds needed to reach the targets. Thus, the system can establish a percentage score of the performance obtained for each exercise. The protocol of instrumental evaluation included two different sets of exercises: Weight Shifting Activity and Mobility Activity.

In the Weight Shifting Activity (Figure 1), patients were asked to move their body axis with variable directions in space, keeping their feet still on the platform. Trials consisted of right, left, back, forth and circles at different speeds and amplitude. Each series consisted of six exercises, two of which were performed on a soft support surface (pillow). The difficulty of the trials was progressively increased. Once a level was reached, the next level was suggested with the visual target, corresponding to the patient’s movement, of greater speed and amplitude. In the Mobility Activity (Figure 2), patients performed six different exercises in movement, with progressively increasing difficulty and speed, in various anteroposterior and lateral directions.

Finally, the home exercises consisted of four series of different exercises for the maintenance of static and dynamic posture of progressively increasing difficulty, eye movements and aim tracking of different types.

After rehabilitation treatment, the Sensory Organization Test (SOT) and DHI were evaluated again. Data obtained before and after therapy were statistically analyzed. Results are presented as means ± standard error of the mean (SEM) and differences were assessed by using variance analysis ANOVA (Statistica, Statsoft, Tulsa, OK, USA); *p* value < 0.05 was considered significant.

## 3. Results

Notwithstanding the limited sample size, the three populations were found to be adequately homogeneous. Specifically, the variance among the groups (*p* = 0.512) and the differences in their means (*p* = 0.735) did not reach statistical significance. A mild facial nerve palsy, classified as House–Brackmann grade II, was observed in only one patient.

Other neurological examination revealed no abnormalities: patients showed no signs of dysmetria during cerebellar testing, and smooth pursuit eye movements were intact.

### 3.1. UVP Group

**Figure 3:**
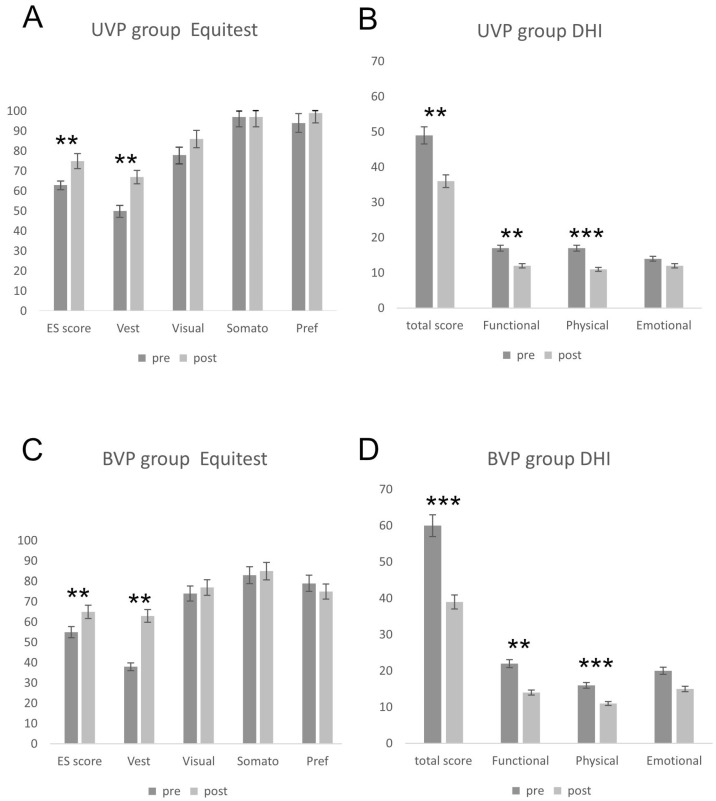
Effects of vestibular rehabilitation on dynamic posturography performance and DHI scores in UVP and BVP groups (** = *p* < 0.01; *** = *p* < 0.001).

In UVP patients (*n* = 19), mean pre-rehabilitation ES was 63 ± 17.39, which significantly improved after rehabilitation treatment (75 ± 10.63; *p* = 0.016). Similarly, vestibular component increased from 50 ± 26.06 to 67 ± 20.61 (*p* = 0.032), while no significant amelioration was detected in visual (78 ± 15.84 vs. 86 ± 11.70; *p* = 0.07), somatosensory (97 ± 5.41 vs. 97 ± 3.94, *p* = 0.93) and preferential (94 ± 18.00 vs. 99 ± 9.04, *p* = 0.31) scores (Figure 3A).

DHI total score improved from a pre-treatment value of 48.84 ± 15.54 to a post-rehabilitation value of 36.21 ± 16.91 (*p* = 0.02). Specifically, a significant amelioration was detected in functional (12.42 ± 6.41 vs. 17.26 ± 7.21, *p* = 0.035) and physical (11.47 ± 5.76 vs. 17.05 ± 5.86, *p* = 0.005) sub-scores, while the emotional score did not improve after treatment (12.42 ± 6.48 vs. 14.53 ± 6.17, *p* = 0.89) (Figure 3B).

### 3.2. BVP Group

All BVP patients enrolled in this study (*n* = 10) showed an abnormal age-matched mean equilibrium score value (55 ± 10.68), which reached a significant improvement after therapy (65 ± 7.16, *p* = 0.02). Similarly to the UVP group, no amelioration was detected in somatosensory, visual and preferential strategy scores, while vestibular component significantly increased from a value of 38 ± 19.74 to a value of 63 ± 24.62 (*p* = 0.03) (Figure 3C).

DHI evaluation showed a significant total score improvement after treatment (39.75 ± 12.89 vs. 60.25 ± 12.11, *p* = 0.005). The functional scale score shifted from 22 ± 5.34 to 14.75 ± 5.84 (*p* = 0.02) and the physical scale shifted from 16.50 ± 4.37 to 11.25 ± 2.37 (*p* = 0.009). No significant changes were detected in the emotional scale pre- and post-treatment scores (21.75 ± 7.88 vs. 15 ± 5.01, *p* = 0.1) (Figure 3D).

### 3.3. PPPD Group

**Figure 4:**
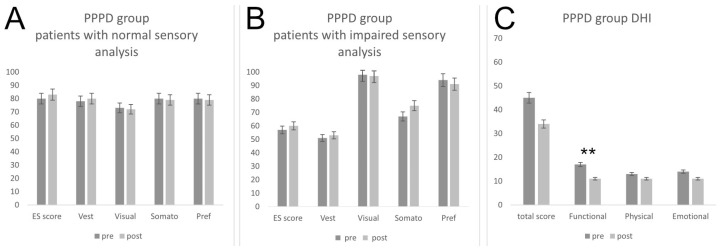
Impact of vestibular rehabilitation on dynamic posturography and DHI scores in PPPD patients (** = *p* < 0.01).

Before treatment, seven patients (47%) showed a normal age-matched mean sensory analysis and equilibrium score, which was not significantly improved after rehabilitation (80.71 ± 5.08 and 83.01 ± 3.57 respectively, *p* = 0.322) (Figure 4A). The remaining patients (*n* = 8) showed a slight pre-treatment ES alteration (57.01 ± 7.74) with vestibular impairment (51.61 ± 17.03) and no somatosensory (98.25 ± 9.48), visual (66.65 ± 12.20) and preferential strategy alterations (94.20 ± 22.24), which was not improved after rehabilitation (ES = 60.05 ± 8.5, *p* = 0.59) (Figure 4B).

With regard to DHI evaluation at admission, all patients (*n* = 15) showed an abnormal DHI total score, of about 45.564 ± 18.21, which was not significantly ameliorated after rehabilitation, reaching a value of 34.13 ± 12.22 (*p* = 0.055). The post-treatment functional sub-score showed a mean value of 11.6 ± 5.35, which was significantly lower as compared to a pre-treatment value of 16.936 ± 7.95 (*p* = 0.04). In contrast, no significant amelioration was detected with regard to the other sub-scores, as the physical scale shifted from a pre-treatment score of 13.6 ± 6.64 to a post-rehabilitation value of 11.6 ± 4.85 (*p* = 0.35) and the emotional scale pre- and post-treatment scores reached a mean value of 14.8 ± 7.43 and 10.8 ± 3.98, respectively (*p* = 0.077) (Figure 4C).

The different responses found in the three groups highlighted the need for personalized rehabilitation strategies for each patient.

## 4. Discussion

The first result of our study indicates that vestibular rehabilitation ameliorates quality of daily life activities, reducing the level of self-perceived handicap both in patients affected by unilateral or bilateral vestibular impairment and PPPD ones. We highlighted that, in all patients, the global score and the functional aspects investigated by the DHI-I significantly improve. As described by Nola et al. [12], the functional score evaluates the interference of dizziness on the performance of movements of the eyes, head and body, thus examining the ability to carry out professional, domestic, social and leisure activities, as well as independence in walking without support and in the dark.

Moreover, in patients with unilateral vestibular deficit (UVP group) we also showed the improvement in the DHI for the physical component, which evaluates the relationship between the presence and/or severity of dizziness and eye or body movements [12]. In this group, we also demonstrated an improvement in global postural control (equilibrium score) and vestibular afferences at the sensory analysis using dynamic stabilometry. These functional and clinical results agree with many data in the literature [14,15] suggesting on the basis of strong evidence that vestibular rehabilitation effectively reduces the frequency of dizziness in adults with various unilateral peripheral vestibular disorders, improving functional activities, and quality of life [16]. In these patients, the rationale of vestibular rehabilitation and its great effectiveness is related to the promotion of vestibular compensation, which is divided into two phases: static and dynamic. Static vestibular compensation is divided into initial and late processes. Rehabilitation acts primarily to facilitate dynamic vestibular compensation in patients with uncompensated unilateral vestibular dysfunction [17]. Central compensation implies three main mechanisms, namely adaptation, substitution and habituation. The compensation, aided by the rehabilitation, aimed to compensate and/or to correct the underused or misused visual, proprioceptive and vestibular inputs involved in the postural control. These pathophysiological considerations therefore explain our results about the improvement of global postural control and the related scores of vestibular components. Finally, our results using Balance Master training are in agreement with the experience of David et al. [3], who evaluated the effectiveness of rehabilitation using vestibular retraining using computerized dynamic posturography associated with home rehabilitation sessions in 13 patients with bilateral vestibular deficit.

In contrast of unilateral vestibular dysfunction, few studies have documented the impact of vestibular rehabilitation on the recovery rate of patients with bilateral vestibular hypofunction, showing different results [18,19,20]. However, according to the American Physical Therapy Association’s clinical practice guidelines, patients with bilateral vestibular disfunction can profit through VR [15]. A recent paper by Genç et al. [21] studied the effectiveness of vestibular rehabilitation on the severity of dizziness, kinesiophobia, balance, fatigue, sleep quality, activities of daily living and quality of life in subjects with bilateral vestibular deficit, showing the enhancement of referred symptoms and quality of life. Similar to patients with unilateral hypofunction in our series, patients with bilateral vestibular deficit also benefit from VR with an improvement both in DHI (functional and physical components) and in equilibrium score and vestibular inputs evaluated by dynamic stabilometry. In these patients, we can hypothesize that, although vestibular compensation is not expected, vestibular rehabilitation with a sensory substitution strategy can improve the imbalance [17].

However, one of the most interesting and innovative results of our study concerns the improvement of patients in the PPPD group. Our results relating to the improvement in the DHI are in agreement with the literature [8,22]. Although rehabilitation does not significantly affect posturographic performances, it ameliorates quality of daily life activities, reducing the level of self-perceived handicap.

Unlike patients with peripheral vestibular deficit, in PPPD, the goal of therapy is not to promote central compensation but to reduce maladaptive postural control strategies and abnormal sensory integration. The association between PPPD and increased visual dependence, impaired sensory reweighting, and impaired spatial orientation processing is known, resulting in instability, discomfort, and anxiety, especially in dynamic or visually complex environments [4,6,7]. In these patients, we hypothesize that rehabilitation works by facilitating habituation and improving the integration of vestibular, visual, and somatosensory inputs. Exposure to progressively more complex visual and motor stimuli may reduce hypersensitivity to movement signals and promote a more automatic and adaptive postural control strategy [8].

With our rehabilitation protocol based on dynamic posturography, patients performed training activities that required continuous adjustment of body position using visual feedback, shifting their weight to reach targets displayed on the monitor. This type of training can improve sensory reintegration and spatial orientation control, which could explain the improvement in DHI scores observed in our PPPD patients even in the absence of significant changes in balance scores.

A recent Cochrane Library review highlighted that responses to vestibular rehabilitation vary widely between individuals [23] and different efficacy could be due to various VR techniques and associations with pharmacological and/or psychotherapeutic treatments. VR using our technique is uncommon, although it has shown promise in vestibulopathy [2,3] and in Parkinson disease [24]. Despite our encouraging data, treatment of PPPD remains controversial. Medical treatment and psychotherapy provide good symptom control, but they can have uncomfortable side effects and short-term relief, respectively [8]. On the other hand, virtual reality customized to the individual’s needs to provide adequate symptom relief without side effects can improve balance and reduce dizziness; however, the modalities and duration remain to be established.

A final consideration on our results with the Balance Master training system concerns patient compliance. Poor adherence to vestibular rehabilitation protocols is a known barrier to optimal care and [25] compliance in VR certainly has an enormous effect on the results; with our technique, the immediate feedback makes the patient more involved in the rehabilitation training. They immediately perceive the improvement, and the progressive increase in exercise difficulty plays a fundamental role in their self-perception, stimulating ever greater participation.

The present study has several limitations that should be considered when interpreting the results. First, no psychological assessment was performed. Standardized psychological scales were not included because the primary aim of the study was to evaluate the effects of vestibular rehabilitation on postural parameters and perceived handicap.

Second, the sample size, particularly in the PPPD group, was relatively small. The limited number of patients may reduce the statistical power of the analysis and could partially explain the lack of significant changes in posturographic parameters. Larger studies are required to confirm the present findings and to better define the effects of vestibular rehabilitation in PPPD patients, including the possible interaction between psychological status and rehabilitation outcomes.

Finally, the absence of a control group represents another limitation of this study. However, controlled trials in PPPD are still limited, and several reports in the literature are based on small observational series or pre–post-treatment designs [7,8]. Recent reviews have highlighted the lack of high-quality controlled studies in this field, making it difficult to draw definitive conclusions about the efficacy of different therapeutic approaches [23]. For these reasons, the present results should be considered preliminary and need confirmation in larger, controlled studies with longer follow-up.

Despite these limitations, we can suggest this rehabilitation technique both in patients with vestibular deficit and in patients suffering from PPPD. Our results support the idea of personalized medicine in vestibular rehabilitation, tailored to the individual characteristics of each patient.

## 5. Conclusions

We confirm that vestibular rehabilitation is effective in unilateral and bilateral peripheral vestibulopathies. In patients with PPPD, rehabilitation performed with dynamic posturography may reduce the perceived handicap and improve some aspects of daily functioning, although the limited sample size and the absence of a control group do not allow definitive conclusions about its efficacy.

Patients who cannot undergo medical therapy and who have difficulty undertaking psychological therapy still have a chance of improving with this treatment modality.

Finally, the Balance Master training system with immediate visual feedback improves compliance, making the patient more involved in the training and improving outcomes. In addition, further clarifying the differential outcomes of vestibular rehabilitation according to specific pathological conditions and patients will pave the way for the development of ever more precise and personalized treatment protocols.

## Figures and Tables

**Table 1:** Main characteristics of study groups.

	UVP	BVP	PPPD
Patients	19 (11 M, 8 F)	10 (4 M, 6 F)	15 (6 M, 9 F)
Age	45–80 years	49–80 years	28–82 years
Mean age	64.4	65.2	61.8
Depression	1/19	0/10	7/15
Anxiety	3/19	3/10	10/15
Falling	2/19	3/10	3/15
Main comorbidities (*n* of patients)	Hypertension 7 Diabetes mellitus 6 Benign prostatic hyperplasia 3 Migraine 2 Hypothyroidism 2 Hyperuricemia 1 Disc herniations 2	Hypertension 3 Diabetes mellitus 4 Migraine 2 Tricuspid valvulopathy 1 Strabismus 1 Tricuspid valvulopathy 1	Hypertension 4 Diabetes mellitus 4 Migraine 5 Cervical spondylosis 1 Chronic venous insufficiency of the lower limbs 1 HCV-related hepatopathy 1
Overall Vestibular assessment	Main findings consistent with unilateral areflexia	Main findings consistent with bilateral areflexia	-
Crebellar function tests	Normal 19/19	Normal 10/10	Normal 15/15
Testing for dysmetria	Normal 19/19	Normal 10/10	Normal 15/15
Pursuit	Normal 18/19	Normal 10/10	Normal 15/15
Facial nerve evaluation	Normal 18/19	Normal 10/10	Normal 15/15

UVP: unilateral vestibular vestibulopathy; BVP: bilateral vestibular vestibulopathy; PPPD: Persistent Postural–Perceptual Dizziness; HCV: Hepatitis C Virus.

## Data Availability

The original contributions presented in this study are included in the article. Further inquiries can be directed to the corresponding author.
